# The Free Association Task: Proposal of a Clinical Tool for Detecting Differential Profiles of Semantic Impairment in Semantic Dementia and Alzheimer’s Disease

**DOI:** 10.3390/medicina57111171

**Published:** 2021-10-28

**Authors:** Gian Daniele Zannino, Roberta Perri, Camillo Marra, Gulia Caruso, Matteo Baroncini, Carlo Caltagirone, Giovanni Augusto Carlesimo

**Affiliations:** 1 Laboratory of Clinical and Behavioural Neurology, I.R.C.C.S. Santa Lucia Foundation, 00179 Rome, Italy; Roberta.perri@tiscali.it (R.P.); g.caruso@hsantalucia.it (G.C.); baroncini.m88@gmail.com (M.B.); c.caltagrone@hsantalucia.it (C.C.); memolab@hsantalucia.it (G.A.C.); 2 Department of Neuroscience, Catholic University of the Sacred Heart, 00168 Rome, Italy; camillo.marra@unicatt.it; 3 Memory Clinic, Policlinico Agostino Gemelli Foundation, I.R.C.C.S., 00168 Rome, Italy; 4 Department of Psychology, University of Rome Sapienza, 00185 Rome, Italy; 5 Department of Neurology, University of Rome Tor Vergata, 00133 Rome, Italy

**Keywords:** semantic dementia, Alzheimer’s disease, semantic memory, free associations

## Abstract

*Backround and Objectives*: It is widely agreed that patients suffering from Alzheimer’s disease (AD) and patients suffering from semantic dementia (SD) might fail clinically administered semantic tasks due to a different combination of underlying cognitive deficits: namely, degraded semantic representations in SD and degraded representations plus executive control deficit in AD. However, no easy administrable test or test battery for differentiating the semantic impairment profile in these populations has been devised yet. *Materials and Methods*: In this study, we propose a new easy administrable task based on a free association procedure (F-Assoc) to be used in conjunction with category fluency (Cat-Fl) and letter fluency (Lett-Fl) for quantifying pure representational and pure control deficits, thus teasing apart the semantic profile of SD and AD patients. *Results*: In a sample of 10 AD and 10 SD subjects, matched for disease severity, we show that indices of asymmetric performance contrasting F-Assoc and each of the two verbal fluency tasks yield a clearly distinguishable discrepancy pattern across SD and AD. We also provide empirical support for the validity of an asymmetry measure contrasting F-Assoc and Cat-FL as an index of control impairment. *Conclusions*: The present study suggests that the free association procedure provides a pure measure of degradation of semantic representations avoiding the confound of possible concomitant executive deficits.

## 1. Introduction

Probably, most of our successful interactions with the environment rely on some kind of explicit or implicit conceptual knowledge [1]. The centrality of semantics in human mental activity is perhaps the reason why no single neuropsychological test is well suited for assessing semantic memory unconfounded with the status of other cognitive systems. Therefore, failing in the same semantic task does not necessarily entail having the same underlying cognitive deficit. This point is rather obvious when we consider the possible contribution of, say, visual processing in a commonly used “semantic task” like picture naming, or that of orthographic input lexicon in matching a written word with the corresponding definition. To tease apart the role of semantic representations deficits from that of impaired “vertical systems” [2] when assessing semantic memory is, however, a relatively simple endeavour, since we can administer a further semantic task to our patient that does not require the putatively impaired domain-specific extra semantic system. Thus, for example, if we suspect that a patient failed the picture naming task due to an impairment in his visual processing system, we can compare performance on this task with that on another semantic task not requiring visual processing (e.g., naming to definition).

Much more challenging is any attempt to disentangle the impairment of semantic representations from the inability to manipulate them for task-specific goals. There are several hypotheses pointing to the need for some kind of “horizontal” [2] executive control resources in addition to intact semantic representations in order to correctly deal with semantic tasks. The old distinction between access and storage deficits in Alzheimer’s disease is a reminder of this issue. According to Nebes [3], persons suffering from Alzheimer’s disease are not able to consciously access preserved semantic representations as demonstrated by impaired picture naming despite preserved semantic priming. More recently, in the framework of controlled semantic cognition [4], new insight in the possible distinction between the integrity of semantic representations and the ability to correctly deal with them has been provided.

The issue of distinguishing the role of semantic representations from that of control resources in solving commonly used semantic tasks is of special interest, when contrasting the cognitive profile of SD and AD. SD is one of the clinical entities associated with frontotemporal lobar degeneration [5]. In this syndrome, although other cognitive functions may become affected as the disease progresses, language remains the most impaired domain throughout the course of the illness. Particularly, anomia and single-word comprehension deficits are the core features in SD, while other levels of language organization like phonology and grammar are not affected [6,7]. A similar pattern of language impairment is often observed in AD although, in this case, deficits in non-linguistic domains (particularly episodic memory) are on the foreground [8]. It is widely agreed that in both pathologies, language symptoms result from impaired semantic knowledge; however, it has been proposed that SD and AD might be characterized by different patterns of disproportionate “representative” and “control” impairments. In SD, a relatively pure deficit for semantic representations has been proposed [6,7,8]. By contrast, in addition to a representational deficit, a major role for disrupted control resources has been advocated as the underlying cause of the observed semantic failures in AD [9].

Recently, it has been proposed that the analysis of asymmetric performance patterns in two paired fluency tasks (namely, letter and category fluency) might provide useful insights in the underlying causes of impaired performance in widely used semantic tasks [10,11]. In both fluency tasks, participants are required to produce as many words as possible under time constraint. In letter fluency (Lett-Fl), the words to be produced have to begin with a given letter (e.g., F), while in category fluency (Cat-Fl), they should belong to the same semantic category (e.g., animals). The rationale of this method bases on the assumption that Lett-Fl relies primarily on control resources, posing only minor requests to the integrity of semantic representations. Cat-Fl, by contrast, still taxing executive resources, is thought to rely mainly on the availability of intact semantic representations. Accordingly, a more severe impairment in Cat-Fl than Lett-Fl might suggest a major impairment of semantic representations over control resources. Unfortunately, however, despite the fact that the semantic deficit in AD and SD is thought to be characterized by a different contribution of control and representative deficits (see above), this method has proven to be unsuitable for reliably distinguishing between these two patient groups, as both populations exhibit very similar patterns of disproportionate impairment for Cat-Fl [8,12].

In an attempt to improve the clinical tools available for distinguishing between deficits of representation vs. control resources in patients showing reduced performance in semantic tasks, we propose and verify the suitability of a new, easily administrable task expressly devised in the present study for capturing deficits of semantic representations while posing extremely low demands on the control system. This task consists of a free association procedure based on a set of cue words for which associative norms have been collected [13]. It is assumed that the relationships between a cue word to its associate is semantic in nature (At least for words with high associative agreement, such as those included in our test (see Section 2.2.1). High associative agreement qualifies those words for which the normative samples produce relatively few different associates with relatively high associative strength. The reader is referred to [13] for a more in-depth discussion of our claim about the semantic nature of associative links) and that, when semantic representations are intact, the production of an associate word in response to a cue word occurs in an automatic manner, without the need of control resources. The procedure of our F-Assoc task is very simple. Participants are presented with cue words to which they have to respond with the first word that comes to mind. Their responses are then scored according to the percentage normative controls which gave the same response [13]. High scores indicate that the participant is generating words produced by a high number of healthy participants, which in turn suggests the integrity of her/his semantic representations. By contrast, idiosyncratic or rare responses give rise to low scores (none ore only a few of the subjects in the normative sample gave the same responses) which are suggestive of degraded semantic representations.

Before detailing the more specific aims of this work and the way we will pursue them, we would like to recapitulate and further qualify the theoretical framework in which it is set. There are three general assumptions upon which this project is based. These are as follows: (i) SD and AD own different cognitive profiles in terms of the relative impairment of control vs. representational resources underlying their semantic deficits; (ii) representational vs. control resources are involved to a variable extent in different tasks sensitive to semantic impairment; (iii) within the same semantic task, different items might require the relative contribution of representations vs. control resources to a different extent in a predictable way, depending on particular features inherent to the task’s items. The first and the second point have been already introduced above. Next, we will better detail both of them, and we will address the third one with respect to two commonly used semantic tasks included in this study, i.e., the pyramids and palm trees test [14] and picture naming.

As we have already seen, it is well known that people suffering from SD exhibit a more severe and at the same time more pure semantic deficit than people suffering from AD. This latter group of patients, indeed, is usually less impaired in semantic tasks while exhibiting poorer performance in a range of extra-semantic tasks taxing episodic memory, visual processing and, more importantly in the present context, executive function resources [8,12,15,16].

Regarding the second point, two kinds of semantic tasks were employed in this study: diagnostic and semantic tasks. Diagnostic tasks, including F-Assoc, Cat-Fl and Lett-Fl, are so termed because they are deemed suitable for investigating the status of control resources and semantic representations in SD and AD patients. To this end, diagnostic tasks are complemented with indices of performance discrepancy, which are also thought to be suitable for quantifying representational or control impairments (see below). As we reported above, a large body of literature claims that Cat-Fl taxes the integrity of semantic representations more heavily than Lett-Fl, while both tasks rely on control resources to a comparable extent [8,10]. In a similar vein, we claim that our newly devised F-Assoc task has the merit of being less demanding in terms of control resources as compared to Cat-Fl (and even more so with respect to Lett-Fl), while being equally demanding in terms of intactness of semantic representations. The semantic tasks used in the present study are two clinically widely used tasks for assessing semantic memory deficits. They comprise a slightly modified form of the picture version of the pyramids and palm trees test (mPPT) and the picture naming test (PN) included in the battery of tests by Catricalà and colleagues [17]. Here, we argue that while both these tasks rely to the same extent on intact semantic representations, PN poses only minor requests on the control system [11] while the PPT also requires considerable control resources in order to distinguish between relevant and irrelevant semantic information present in the depicted items.

Coming to the third point, i.e., to the item-specific variables argued to modulate the relative requirements of control and representative resources, it is plausible that the amount of control resources needed to manipulate semantic information in the PPT varies across items, as isolating the critical semantic information linking the reference figure to the target figure might be more or less intuitive. We argue that, since items requiring more control resources are probably also more time consuming, items mean reaction times (RTs) registered in an unimpaired population could be a reliable proxy of reliance on control resources. As a consequence, we expect RT to modulate the effects of control impairment on PPT accuracy.

As for PN, it is well known that patients suffering from SD are more accurate when naming high-frequency and high-familiarity words presumably because their semantic representations are more robustly encoded. On the other hand, it has been proposed that high-frequency/-familiarity items are particularly demanding for control resources. The rationale beyond this claim is that high-frequency words, due to their use in many different contexts, have a higher degree of polysemy, which require an accordingly high level of control function to select the relevant meaning for the task at hand [18]. In keeping with this claim, the scarce impact of word frequency in patients suffering from semantic aphasia, at variance with the protective effect of high word frequency observed in SD, has been explained with a deficit of control resources, in addition to that of semantic memory, in this vascular syndrome [4].

In the present study, we administered all of the above-mentioned diagnostic and semantic tasks to a group of 10 AD patients and 10 SD patients. Patients were matched for mini-mental state examination score (MMSE [19]) in order to control for disease severity/global cognitive impairment [8,11,12]. The collected data were used with a twofold aim: first, to compare the clinical suitability of F-Assoc, Cat-Fl, Lett-Fl and derived discrepancy indices for teasing apart SD and AD cognitive profiles and, second, to verify the above listed predictions about the validity of discrepancy scores across pairs of diagnostic tasks as indices of disproportionate impairment in representational vs. control resources.

## 2. Materials and Methods

### 2.1. Participants

All experimental tasks were administered to a group of 10 participants suffering from AD and a group of 10 participants suffering from SD. AD patients received the diagnosis of AD based on the clinical criteria established by the National Institute of Neurological and Communicative Disorders and Stroke/Alzheimer’s Disease and Related Disorders Association [20]. SD patients were diagnosed according to the guidelines published by Gorno-Tempini and colleagues [6]. All patients underwent an evaluation by a team of neurologists and psychologists who were experts in dementia. Their medical history, neurological examination, brain imaging and laboratory tests confirmed that their dementia symptoms could not be attributed to an illness other than AD/SD.

All of the tasks except PN were also administered to a group of 25 healthy seniors to serve as control. Finally, a group of 50 young adults was recruited for collecting normative data on mean RT of the mPPT (see below).

Although all of the recruited patients underwent an extensive neuropsychological examination at the time when the diagnoses were posed, a variable period of time elapsed from that moment to the time when the experimental tasks were administered. Therefore, data referring to the background neuropsychological evaluation were not reported, as they did not necessarily reflect the cognitive profile of the patients at the time when the experimental investigation took place. At that time, only MMSE was repeated to obtain an up-to-date measure of disease severity [8,12]. The absence of an up-to-date background examination is a limitation of the present study that we want to acknowledge now. On the other hand, we also want to draw the reader’s attention to the fact that AD and SD were matched on a one-to-one basis for MMSE. Such a strict matching procedure represents an unusual opportunity to make claims about the differential semantic profile of AD and SD not confounded with disease severity.

Table 1 reports the demographic features and MMSE scores of the patients and normal control group (NC). Males and females were equally represented in the two patient groups, while females outnumbered males in the NC group; overall, differences in the distribution of males and females, however, did not reach statistical significance (χ^2^ (2) = 2.6; *p* = 0.271). Healthy controls were comparable for age with AD (F (1,34) = 0.6, *p* = 0.817), while SD patients were reliably younger than both AD (F (1,19) = 19.6, *p* < 0.001) and NC (F (1,34) = 19.0, *p* < 0.001) individuals. The SD group was more educated than AD (F (1,19) = 9.9, *p* = 0.006), while no other pairwise group comparison reached statistical significance (*p* > 0.077, consistently). Finally, age- and education-corrected MMSE scores were perfectly matched between the two pathological groups (F (1,19) = 0.1, *p* = 0.951), while NC significantly outperformed the other groups (*p* < 0.001, for both pairwise comparisons).

All subjects gave their informed consent in accordance with a protocol approved by the Ethics Committee of the IRCCS Santa Lucia Foundation.

### 2.2. Procedures

All the participants underwent the experimental tasks in the same order. First, the three diagnostic tasks were administered starting with F-Assoc, followed by Cat-Fl and ending with Lett-Fl. Then, after a 20 min interval, the semantic tasks were administered, starting with mPPT and ending with PN with a 5 min interval between the two tasks.

#### 2.2.1. Free Associations

The cue words comprising the F-Assoc task and the corresponding normative data stem from a previous study aimed at demonstrating the semantic nature of associative links [13]. The F-Assoc task comprised 35 concrete words to be used as cues in a free association procedure (see below). The words were selected so as to span from an intermediate to a high level of associative agreement. The maximum possible associative agreement occurs when all normative subjects respond with the same associate to a given cue, while the minimum occurs when each subject in the normative sample responds to the same cue with a different word (see [13] for computation details). Each of the 35 cue words is associated with a list of words produced as associates by a group of 52 elderly Italian controls (mean age = 72.9, SD = 7.01, range = 65–89). For each associated word, a measure of associative strength is available, corresponding to the proportion of the 52 controls which generated that word in response to the corresponding cue in the original study [13].

The experimenter spoke each cue word aloud and participants were instructed to respond as fast as possible with the first word that came to mind. Each response was credited with a score corresponding with its associative strength. Thus, for example, the word “rain” in response to the cue “umbrella” was credited 0.8 because 80% of the normative sample in Zannino and colleagues [13] gave the same response; by contrast “water” was credited 0.04 because only 4% of generated this word in response to “umbrella”. Responses never produced by the normative group were credited 0; the same applied for no responses. The total F-Assoc score was calculated as the mean score assigned to the 35 responses.

#### 2.2.2. Category Fluency

In the Cat-Fl task participants had to generate as many words belonging to a given category as possible within 1 min. The task comprised three categories: animals, fruits and vehicles; the categories were administered in this order to all the participants. The total Cat-Fl score was calculated as the sum of correct responses across the three categories.

#### 2.2.3. Letter Fluency

In the Lett-Fl task, participants were asked to generate as many words beginning with a given letter as possible within 1 min. The task comprised the letters F, A and S in this order. The total Lett-Fl score was calculated as the sum of correct responses across the three letters.

#### 2.2.4. Pyramids and Palm Trees

The PPT [14] is a test of semantic association. In the visual version of the PPT, participants are presented with three pictures arranged at the vertices of a triangle. The participant had to select which picture of the two alternatives depicted at the base of the triangle better matched the picture at the top of the triangle.

In the present study, a modified form of the visual PPT task was used (mPPT). The present form comprised colour photographs instead of the original line drawings. This change aimed at reducing possible confusion due to the fact that the original line drawings are sometimes outdated and therefore not easy to recognize. Since we were interested in collecting reaction times as a measure of the amount of control resources required by each single task item, it was mandatory to prevent noise deriving from unequally long visual processing times across test items. In order to facilitate visual recognition, care was taken to select photographs depicting typical tokens of each concept in a canonical view.

To collect reaction times, a computerized version the mPPT was administered on a PC controlled by E-Prime software (version 3) to a group of 50 healthy young adults (age in years: mean (SD)/range = 23.8 (2.7)/19–30; education in years: 15.3 (1.8)/13–18). The participants sat at 60 cm from a 15-inch computer monitor in a dimly lit room; they were instructed to select the picture on the left/right bottom site of the screen as fast and accurately as possible by pressing with their dominant hand, respectively, the K and L keys on the keyboard. After removing RT to incorrect responses and outliers (+/−2SD), mean reaction times for the 52 items of the mPPT were computed to serve as the predictor variable in subsequent analyses.

The mPPT was administered to SD, AD and NC without time constraint; responses were scored either as correct (1) or incorrect (0).

#### 2.2.5. Picture Naming

The 48 coloured photographs comprising the PN test of the semantic battery devised by Catricalà and co-workers [17] was administered to AD and SD on a computer screen. Responses were scored as either correct (1) or incorrect (0). Log transformed word frequency (W-freq) values were available for the included items [21].

#### 2.2.6. Statistical Analyses

The analyses reported in the results sections and the corresponding plots were carried out using R version 4.0.3 [22].

Our first aim was that of comparing the clinical suitability of our diagnostic tasks (i.e., F-Assoc, Cat-Fl and Lett-Fl) for teasing apart SD and AD cognitive profiles. To this end, we performed three separate Kruskal–Wallis tests to compare the performance across the three participant groups on each task. We further qualified the significant results with pairwise comparisons using Wilcoxon rank-sum tests (see Section 3.1). In a second step (see Section 3.2), following Crawford and colleagues [23], for each participant, a discrepancy score was computed for each pair of diagnostic tasks (i.e., Lett-Fl vs. Cat-Fl, Lett-Fl vs. F-Assoc and Cat-Fl vs. F-Assoc) as a point estimate of the effect size of the difference between the two tasks. Discrepancy scores were then analysed using the same non-parametric tests used in Section 3.1.

A second aim of the present study was that of verifying the validity of discrepancy scores across pairs of diagnostic tasks as indices of disproportionate impairment in representational vs. control resources. To this end (see Section 3.3.1 and Section 3.3.2), data collected in our semantic tasks (i.e., mPPT e PN) were used. We performed several analyses using mixed logistic models with crossed random factors for subjects and items (R package lme4 [24]). Responses to each single task item, binary encoded as either “correct” or “incorrect”, were entered as dependent variables. In a first step, we investigated the relative suitability of F-Assoc and the other diagnostic measures (i.e., Cat-Fl and Lett-Fl) in predicting performance on the two semantic tasks. In a second step, we investigated the interactions of a subject-specific control function index (based on the relative proficiency in Cat-Fl vs. Free-Assoc) and of Lett-Fl (a well-known measure of control function) with two item-specific variables of mPPT and PN (i.e., RT and word frequency, respectively) deemed to be reliably associated with the degree of reliance on control functions (see Introduction).

## 3. Results

### 3.1. Diagnostic Tasks

**Figure 1:**
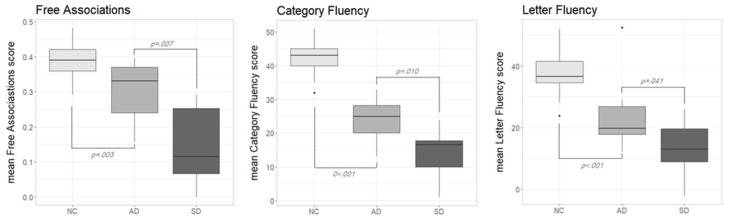
Boxplots showing the performance in the three diagnostic tasks according to participants group. AD—participants with Alzheimer’s disease, NC—normal controls, SD—participants with semantic dementia.

Figure 1 shows the performance in the three diagnostic tasks according to the different participants groups (NC, AD and SD). Lett-FL scores were adjusted for age and education [25]. Normative data for F-Assoc and Cat-FL were not available; thus, raw data were used. As can be seen, in all of the diagnostic tasks, NC outperformed both pathological groups, while AD consistently outperformed SD. The visual inspection of the boxplots on Figure 1 was confirmed with a series of non-parametric tests.

In the F-Assoc task, the Kruskal–Wallis test confirmed significant performance differences across the three participants groups (H (2) = 26, *p* < 0.001). Post hoc comparisons with Wilcoxon rank-sum tests showed significant differences between NC and AD (*W* = 42.5, *p* = 0.003, *r* = 0.506) as well as between AD and SD (*W* = 86, *p* = 0.007, *r* = 0.600). Obviously, also the SD-versus-NC comparison yielded a significant difference (*W* = 249, *p* < 0.001, *r* = 0.762).

A similar pattern applied to the Cat-Fl task: the Kruskal–Wallis test was statistically significant (H (2) = 34, *p* < 0.001) as well as all of the follow-up tests; NC vs. AD: (*W* = 2, *p* < 0.001, *r* = 0.757); AD vs. SD: (*W* = 85, *p* = 0.010, *r* = 0.575); SD vs. NC: (*W* = 250, *p* < 0.001, *r* = 0.770).

Finally, for Lett-Fl, the overall performance level significantly differed across groups: Kruskal–Wallis test (H (2) = 25, *p* < 0.001). AD scored significantly poorer than NC (*W* = 25, *p* < 0.001, *r* = 0.615); the same was true for the SD vs. NC comparison (*W* = 243, *p* < 0.001, *r* = 0.730). A reliable difference was also observed between AD and SD (*W* = 78, *p* = 0.041, *r* = 0.457).

Summarising the results of these analyses, we can say that the finding that AD outperformed SD in F-Assoc and Cat-Fl is expected on the widely agreed assumption that, when disease severity is comparable (as it is the case in our samples), SD exhibits a more severe semantic deficit than AD. It should be noted, however, that Cat-Fl also relies on control resources, thus the interpretation of the observed cross groups pattern is far less straightforward than for F-Assoc. Finally, the finding that SD were at a disadvantage also in Lett-Fl is unexpected, since AD patients are supposed to suffer a greater control impairment than SD patients. We will return to these issues in the discussion section (It should be noted that AD patients were older and less educated than SD ones, thus the disadvantage of SD in F-Assoc and Fl-Cat is probably somewhat underestimated).

### 3.2. Performance Asymmetries in Diagnostic Tasks

As remembered in the introduction, a disproportionate impairment in Cat-Fl as compared to Lett-Fl has been often interpreted as the hallmark of a semantic memory deficit. We were interested in comparing this discrepancy measure with those involving the newly devised F-Assoc measure in our pathological samples. To this end, we computed three different discrepancy scores for each participant in the AD and SD groups, based on the performance of the NC group. Following Crawford and colleagues [23], the discrepancy in two paired tasks was expressed as a point estimate of the effect size of the difference between the two tasks. This index takes the value 0 when the performance is comparable across tasks and assumes growing positive or negative values when a subject exhibits a disproportionate impairment on the first or second task, respectively. The software provided by Crawford and colleagues [23] also computes the *p* value associated with the observed dissociation at the individual level.

**Figure 2:**
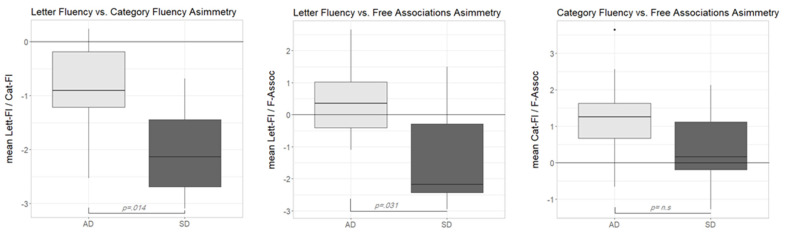
Boxplots showing the distribution of the asymmetry scores according to the participant groups. AD—participants with Alzheimer’s disease, NC—normal controls, SD—participants with semantic dementia.

As can be seen in Figure 2 (left plot), both participant groups showed negative asymmetry scores in the Lett-Fl vs. Cat-Fl comparison. This result confirmed previous reports of a disproportionate impairment in Cat-Fl as compared to Lett-Fl in both populations. A Wilcoxon signed rank test showed that in both the AD group (*V* = 5, *p* = 0.020, *r* = 0.738) and the SD group (*V* = 0, *p* = 0.002, *r* = 0.979), asymmetry scores were reliably different from zero. Moreover, a Wilcoxon rank-sum test showed that the median discrepancy score was reliably lower in the SD than in the AD group (*W* = 83, *p* = 0.014, *r* = 0.549), indicating a stronger dissociation in the former group. At the single-subject level, a reliable dissociation was observed nine times (three participants were AD and six SD).

The middle plot in Figure 2 shows the distribution of the asymmetry scores of the Lett-Fl-versus-F-Assoc comparison across groups. In this case, the median scores were reliably different across groups (Wilcoxon rank-sum test: *W* = 79, *p* = 0.031, *r* = 0.481). Interestingly, however, this time, the pattern of disproportionate impairment was not the same across groups. While the median asymmetry score of the AD group was unreliably above zero (*V* = 37, *p* = 0.375, *r* = 0.281), suggesting the substantial absence of any dissociation across tasks in this group, the median discrepancy score in the SD group was reliably below zero (*V* = 7, *p* = 0.037, *r* = 0.659), suggesting a major impairment in F-Assoc as compared to Lett-Fl. At the single subject level, seven reliable dissociations were observed. One participant in the AD group was reliably more impaired in Lett-Fl as compared to F-Assoc, while six SD participants showed the opposite impairment pattern (better performance on the Lett-Fl task).

Finally, the right plot in Figure 2 shows the asymmetry scores according to the two participant groups in the Cat-Fl-versus-F-Assoc comparison. This time, asymmetry scores were not reliably different across groups (Wilcoxon rank-sum test: *W* = 68, *p* = 0.186, *r* = 0.296). However, (at variance with the previous comparison) a reliable dissociation (Cat-Fl more impaired than F-Assoc) was only observed in the AD group (*V* = 51, *p* = 0.014, *r* = 0.780). By contrast, the median discrepancy score in the SD group was close to zero, suggesting a comparable impairment across tasks (*V*= 38, *p* = 0.322, *r* = 0.313). At the single-subject level, four reliable dissociations were found (two AD and two SD) with Cat-Fl more impaired in all cases.

Summarizing the results of the discrepancy analyses, we can say that while the classical Lett-Fl-versus-Cat-Fl asymmetry only shows quantitative differences across SD and AD, discrepancy measures involving F-Assoc lead to qualitatively different asymmetry patterns. Indeed, Lett-Fl vs. F-Assoc is only dissociated in the SD group, while the reverse is true (only AD scores are reliably different from 0) for the Cat-Fl-versus-F-Assoc asymmetry. The interpretation of these contrasting patterns will be taken up later on in the discussion section.

### 3.3. Variables Predicting Accuracy in Semantic Tasks

In the previous analyses, we made an attempt to investigate the suitability of the joint use of F-Assoc and two commonly used verbal fluency tasks for individuating differential patterns of impairment in SD and AD. In the following sections we will verify the actual explanatory power of these same variables in predicting SD and AD performance in semantic tasks.

#### 3.3.1. mPPT

Not surprisingly, the performance level on the mPPT was different across the three experimental groups (Kruskal–Wallis test: H (2) = 30, *p* < 0.001). In fact, NC (mean (SD) = 51.1 (0.93)) outperformed both AD (mean (SD) = 46.2 (3.3); Wilcoxon rank-sum test: *W* = 24, *p* < 0.001, *r* = 0.641) and SD (mean (SD) = 38.3 (7.4); *W* = 60, *p* = 0.472, *r* = 0.161). It should be noted that the SD performance could be somewhat overestimated due to unbalanced demographic variables.

Finally, as expected for samples matched for disease severity (as indexed by MMSE), SD scored significantly worse than AD (*W* = 88, *p* = 0.004, *r* = 0.637).

The subsequent analyses were carried out on the data of only the pathological samples (AD and SD). In a first step, we wanted to compare the role of the factor Group (AD vs. SD) with that of our diagnostic variables in predicting mPPT accuracy of brain damaged participants. To this end, participants’ scores on the three diagnostic tasks were transformed into two-level factors assigning 0 to scores below the median split and 1 to scores above the median split. The number of AD/SD participants falling into each level of the diagnostic variables is reported in column 2 of Table 2.

We ran four distinct logistic mixed models (using R package lme4 [24]) entering Accuracy (0 = incorrect, 1 = correct) as a dependent variable, Subject and Item as random factors and each of our categorical predictors (i.e., Group, Cat-Fl, F-Assoc and Lett-Fl) in turn as a fixed factor. Table 2 summarizes the results of these analyses. The different models are displayed from top to bottom in decreasing order of the models’ goodness of fit (−2log likelihood). The column labelled “estimate” reports the log odds associated by the model with the intercept (corresponding to the first level of each predictor; i.e., SD or below median performance on a given diagnostic task) and the changes in log odds when passing from the first to the second level (i.e., AD or above median performance on a given diagnostic task). Since all models only include a binary factor as the fixed predictor, estimates can be directly compared across models as if they were standardized effect size measures. For the sake of clarity, estimated log odds were transformed in predicted probabilities, using the ggpredict function of the ggeffects package [26]. The last three columns report the comparison of each model with a baseline model, containing only the intercept and the two random factors, by means of likelihood ratio tests [27].

As can be seen, all the predictors but Lett-Fl significantly increased the model fit as compared to the baseline model. The factor Cat-Fl turned out to be the best predictor for the performance on the mPPT (followed by Group and F-Assoc) in terms of both effect size (change in log odds) and model fit (−2log likelihood).

In a second step, we wanted to fit a unique logistic mixed model, taking simultaneously into account the factor Group, those diagnostic variables that turned out to be reliably associated to mPPT accuracy in the previous analyses (namely, Cat-Fl and F-Assoc) and the confounding variables of Age and Education. To this end, we fitted a mixed logistic model in lme4, entering Group, Cat-Fl, F-Assoc, Age and Education. This time, however, diagnostic variables were entered as continuous variables, in order to not discard potentially relevant data. Following Barr and co-workers [28], we attempted to include the maximal random effects structure justified by the design. However, the model failed to converge until random slopes were removed. Thus, the final model only included random intercepts for Subject and Item.

Table 3 (Part A) reports the summary of the fixed effects. As can be seen, only Cat-Fl turned out to be reliably associated with accuracy on mPPT. Not surprisingly, the estimate has a positive sign, indicating that increasing performance on Cat-Fl is associated with increasing accuracy on mPPT.

The fact that Cat-Fl had the greater explanatory effect on mPPT performance was expected given the results of the analyses carried out in the first step. More importantly, this result was also expected based on the assumption that both Cat-Fl and mPPT tax the same cognitive resources, i.e., semantic representations and control function, while F-Assoc is thought to be only sensitive to the integrity of semantic representations. By contrast, the fact that Lett-Fl was not reliably associated with accuracy (see Table 2) is somewhat surprising, assuming the involvement of control functions in mPPT. These issues will be taken up later on in the discussion section.

**Figure 3:**
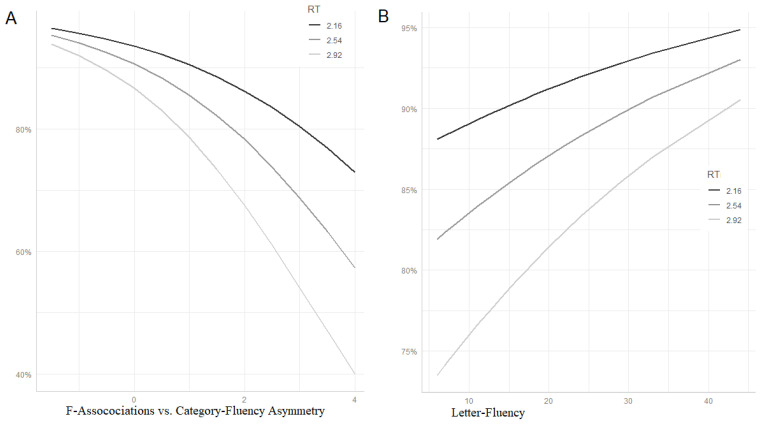
(**A**) Predicted probabilities of correct mPPT according to items’ reaction times in a healthy control group (mean ± SD) and F-associations vs. category-fluency asymmetry (mean ± SD). (**B**) Predicted probabilities of correct mPPT according to items’ reaction times and letter fluency.

In a final step, we wanted to verify whether a disproportionate impairment in Cat-Fl as compared to F-Assoc could be interpreted as the hallmark of a control deficit. To this end, we investigated the interaction between this discrepancy measure and reaction time (RT) in predicting mPTT scores. We reasoned that if disproportionate impairment in Cat-Fl as compared to F-Assoc is suggestive of a control deficit and if mPPT items with longer RT (in normal controls) tax control resources more heavily, then a RT by Cat-Fl-versus-F-Assoc asymmetry (Cat/Free-Asymm) interaction is expected. In particular, we expected that differences in mPPT accuracy between high and low asymmetry scorers (i.e., between people who have or do not have a control deficit, respectively) should be increasingly evident as RT increases. To verify this prediction, we fitted a logistic mixed effect model in lme4, entering RT, F-Assoc and RT by Cat/Free-Asymm interaction as explanatory variables. As a random factor we entered random intercepts for subject and item, by-subject random slopes for RT and by-item random slopes for F-Assoc (i.e., the maximal random effects structure yielding a reliable model [28]). Table 3 (part B) reports the summary of the fixed effects. As expected, both RT and F-Assoc reliably predicted the outcome. Correct responses increased with increasing F-Assoc scores and decreasing RTs. More interestingly for our purposes, a reliable interaction was found. Figure 3 (panel A) uses the ggpredict function of the ggeffects package [26] for visualizing mean predicted accuracy at different values of RT and Cat/Free-Asymm. Lines represent predicted probabilities at different levels of RT (mean ± SD) as a function of Cat/Free-Asymm (range = mean ± SD). As can be seen, high scores in Cat/Free-Asymm (on the right side of the plot), indicative of control deficits, are associated with a disproportionate impairment of slow items (light grey line).

Finally, we wanted to investigate whether the control deficit measured by the Cat/Free-Asymm is identical with the executive function indexed by Lett-Fl. To this end, we fitted another model, otherwise identical to the former, entering Lett-Fl instead of Cat/Free-Asymm (and removing by-item random slopes for F-Assoc to avoid convergence failure). This time, the interaction was only marginally significant (estimate (SE) = 0.011; *z* = 1.668; Pr (>|z|) = 0.095) and the pattern of predicted accuracy for slow items far less influenced by the control variable (see Figure 3, panel B—note that, in contrast to panel A, low executive scores are on the left side of the plot).

Summarising this final series of analyses, we can say that Cat/Free-Asymm seems to be better suited than Lett-Fl as an index of control function affecting the accuracy of our pathological samples on the mPPT.

#### 3.3.2. Picture Naming

The analyses carried out on the PN data strictly followed the approach applied to the mPPT data. In a preliminary step, we only wanted to quantify the level of impairment across the two pathological groups. Since NC did not undergo the PN task, patients’ scores were adjusted for age and education and compared to the 20° percentile cut-off of the normative sample (i.e., a corrected score of 45.82 [17]). A Wilcoxon signed rank test showed that both the AD group (mean (SD) = 43.8 (2.3); *V* = 5, *p* = 0.012, *r* = 0.559) and the SD group (mean (SD) = 25.0 (13.7); *V* = 0, *p* = 0.003, *r* = 0.664) scored reliably below this cut-off. A Wilcoxon rank-sum test revealed that, as expected, SD scored reliably poorer than AD (*W* = 97.5, *p* < 0.001, *r* = 0.795).

In analogy with what we did with the mPPT data, we began by comparing the suitability of our diagnostic variables (i.e., Cat-Fl, F-Assoc and Lett-Fl) with that of the factor Group in predicting PN performance. To this end, we fitted four independent logistic mixed models entering in turn Group and the three diagnostic variables as explanatory factors transformed in binary factors based on the median split (see Section 3.3.1). Subject and Item were modelled as random factors. Table 4 has the same structure as Table 2. Model parameters are displayed in decreasing order of the fitness of the model. This time, the most predictive factor (in terms of both effect size and statistical significance) was Group, followed by F-Assoc and Cat-Fl. In this case, Lett-Fl was not reliably associated with the patients’ performance in the semantic task.

The variables, which turned out to be reliably associated with accuracy in PN, were then entered in a unique logistic model. This time, however, diagnostic variables (Cat-Fl and F-Assoc) were entered as continuous variables alongside Age and Education. Following Barr and co-workers [28], we attempted to include the maximal random effects structure justified by the design. However, the model failed to converge until random slopes were removed. Thus, the final model only included random intercepts for Subject and Item. Table 5 (part A) reports the summary of the fixed effects. In contrast to mPTT, both Cat-Fl and F-Assoc significantly contributed to the model fitness. All other fixed factors, including the confounding variables, fell far from significance.

**Figure 4:**
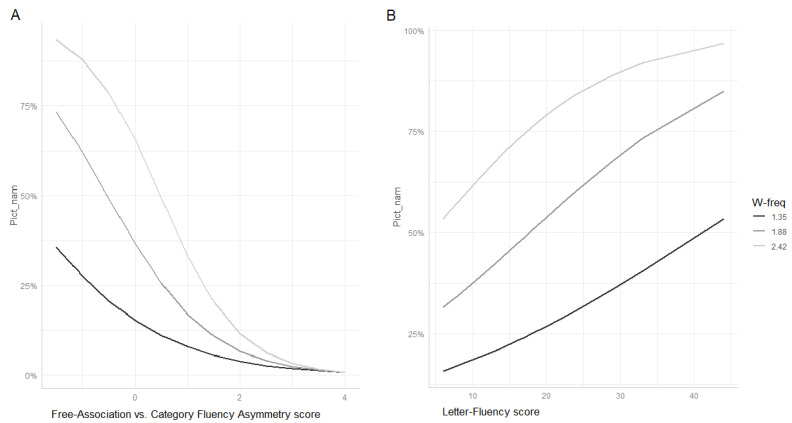
(**A**) Predicted probabilities of correct PN according to word frequency (mean ± SD) and F-associations vs. category-fluency asymmetry (mean ± SD). (**B**) Predicted probabilities of correct PN according to word frequency and letter fluency (the value of the covariate F-Assoc over which the effect is “averaged” and was lowered to −1SD (default = mean) to avoid confusing ceiling effects in the plots).

In a second step, we wanted to investigate whether our index of control deficit (Cat/Free-Asymm) showed a significant interaction with word frequency (W-freq). Based on the assumption that high-frequency words are more taxing for the control system [4], it is expected that people with high asymmetry scores (suggestive of control impairment) do not exhibit any advantage in naming otherwise “easier” high-frequency words. To this end, we fitted a logistic mixed effects model entering W-freq, F-Assoc and W-Freq by Cat/Free-Asymm interaction as explanatory variables. As random factors, we entered random intercepts for Subject and Item, by-subject random slopes for W-freq and by-item random slopes for F-Assoc (i.e., the maximal random effects structure justified by the design [27]). Table 5 (part B) shows a summary of the fixed effects. Both F-Assoc and W-freq were significantly associated to PN accuracy. As expected, accuracy was higher for subjects with high F-Assoc scores and for high-frequency words. More interestingly, W-Freq showed a significant interaction with Cat/Free-Asymm. As can be seen in Figure 4 (panel A), the advantage for high-frequency words decreased with increasing control deficit (high values of Free/Cat-Asymm).

Finally, as we already did with the analysis of the mPPT data, we compared the effect of our control deficit measure (Cat/Free-Asymm) with that of a commonly used index of executive abilities, i.e., Lett-Fl. To this end, we replaced Cat/Free-Asymm with Lett-Fl in the mixed model described above and locked the W-freq-by-Lett-Fl interaction. In contrast to the mPPT data, the interaction was significant (estimate (SE) = 0.035; *z* = 2385; Pr (>|z|) = 0.017). Although far less clear cut, the relationship linking Lett-Fl to W-freq was similar to that between Cat/Free-Asymm and W-freq. In fact, as can be seen in Figure 4 (panel B), the advantage for high-frequency over low-frequency words decreased with decreasing frontal functioning (i.e., low Lett-Fl scores) also in this case.

## 4. Discussion

In this study, we made an attempt to provide empirical evidence supporting the following claims: (i) F-Assoc is a “pure” and reliable measure of degraded semantic representations; (ii) F-Assoc can be used jointly with Cat-Fl and Lett-Fl to reliably differentiate cognitive profiles sustaining poor performance in semantic tasks in SD and AD; (iii) discrepancy between Cat-Fl and F-Assoc is a reliable index of the kind of control function deficit that impacts semantic tasks. To this end, we started from a widely agreed set of claims (with some plausible additions) regarding the cognitive profile of SD and AD persons with respect to representational and control resources, the differential reliance of F-Assoc and verbal fluency tasks on these cognitive resources and the modulatory effect of some item-specific variables (namely, RT and W-freq) on the extent to which two commonly used semantic tasks (PPT and PN, respectively) rely on control function.

We enrolled 10 persons suffering from SD and 10 persons suffering from AD, strictly matched for MMSE. This was done in order to contrast two samples with different pathologies but roughly comparable levels of disease severity and general cognitive functioning [6,11]. This point is important, since comparable levels of disease severity need to be assumed to make reliable claims about the relative proficiency of SD and AD patients in particular cognitive domains (it is obvious, for example, that the claim about a more widespread cognitive impairment in AD would not hold when very mild AD cases are contrasted with very severe SD cases).

Three so-called diagnostic tasks (i.e., F-Assoc, Cat-Fl and Lett-Fl) were administered to our pathological samples (and NC). Starting from the assumption that at comparable levels of disease, SD patients are more impaired than AD ones in representational resources and AD patients are more impaired than SD ones in control resources (see introduction), experimental data were analysed to ascertain if they fitted our claims about the cognitive resources tapped by each single diagnostic task.

As can be seen in Figure 1 (see Section 3.1), most of the results easily fit the set of claims detailed in the introduction. Overall, the AD group outperformed the SD group in the tasks thought to be sensitive to semantic memory impairment (i.e., F-Assoc and Cat-Fl), as expected, assuming a more severe representational deficit in the latter population. It is worth noting that the effect size of the impairment in the AD group as compared to the control group was greater for Cat-Fl as compared to F-Assoc (*r* = 0.757 and *r* = 0.506, respectively). This is expected, assuming that Cat-FL relies on both representational and control resources (both impaired in AD), whilst F-Assoc only relies on the integrity of semantic representations. By contrast, the effect size of the impairment in SD (as compared to NC) is comparable across both tasks (*r* = 770 and 0.762 for Cat-Fl and F-Assoc, respectively), which is in keeping with the assumption of a negligible control impairment in the SD group. Finally, the finding that the AD group also outperformed the SD group in Lett-Fl (although the effect size is much smaller) is, prima facie, in conflict with the assumption of a more defective control function in AD. There is, however, a possible explanation for this result. Indeed, Lett-Fl likely requires little but not no contribution to representational resources, as suggested by Henry and colleagues [11]. Because of this, AD patients could have failed the test due to disproportionately low control resources, while SD patients failed it due to disproportionately low representational resources. In this case, a trade-off effect might have occurred, thus reversing the expected pattern of cross-group dissociation.

Figure 2 (Section 3.2) shows performance asymmetries in pairs of diagnostic tasks across the two pathological groups. The well-known assumption [7,12] of a disproportionate impairment in Cat-Fl as compared to Lett-Fl was confirmed for both populations. It is noteworthy that the pattern is similar across the two pathological samples; thus, this measure seems to be ill suited to reliably tease apart SD and AD. Indeed, although we found that the SD group’s performance was significantly more asymmetric than that of the AD group, this finding is not ubiquitous. For example, Rogers and colleagues [7] found that the disproportionate impairment for Cat-Fl was comparable across MMSE-matched SD and AD groups. These authors correctly argued that a trade-off effect between control resources (disproportionately impaired in AD) and representational resources (disproportionately impaired in SD) can limit the suitability of this measure for distinguishing between SD and AD. A greater discriminative power seems to be ascribable to the other discrepancy patterns reported in Figure 2, namely those involving F-Assoc. When contrasting Lett-Fl with F-Assoc, the AD sample did not show any discrepancy, suggesting that the control deficit (as indexed by Lett-Fl) is as large as the representational deficit measured by Free-ass. By contrast, SD patients showed a clear-cut disproportionate impairment in F-Assoc, as expected in a population with severe representational deficits and negligible control impairments. Finally, also the Cat-Fl-versus-F-Assoc discrepancy pattern fit well in our general framework. First, overall, the observed discrepancy is unidirectional and in the expected direction (i.e., disproportionate impairment for Cat-Fl). In fact, on the assumption that control resources can be useful for solving the task only if semantic representations are sufficiently spared, the only pattern we could expect is that of a disproportionate impairment for Cat-Fl if control resources are poorer than representational ones, while no dissociation is expected in case of a selective disruption of representational resources. Moreover, the finding that SD patients did not dissociate is in keeping with the assumption that the control deficit is negligible in this population. By contrast, the observed disproportionate impairment for Cat-Fl in the AD group suggests that control resources might be more severely affected than representational ones in this population.

In a further series of analyses, we attempted to provide evidence regarding the external validity of our diagnostic tasks and derived asymmetry indices. In a first step, we contrasted the explanatory power of our dichotomized diagnostic measures with the factor Group in predicting mPPT and PN accuracy. The variable which better predicted mPPT accuracy (in terms of both reliability and effect size) was Cat-Fl (see Table 2). This result was expected, based on the assumption that this explanatory variable jointly measures control and representational resources, i.e., exactly the same resources mPPT is assumed to rely upon. By contrast, F-Assoc was the first predictor (in terms of both reliability and effect size, see Table 3) of PN accuracy, which is in keeping with our claim that both tasks rely heavily on representational resources while taxing only marginally control functions.

These analyses were further confirmed and extended with models simultaneously taking in account the effect exerted by all of the explanatory variables (see Table 3A and Table 5A). The major role of Cat-Fl and F-Assoc in predicting accuracy in mPPT and PN, respectively, was confirmed. When diagnostic variables were taken into account, the factor Group was no longer reliably associated with the outcome of our semantic task (i.e., mPPT and PN). This finding suggests that Cat-Fl and F-Assoc actually tap into the cognitive resources which are needed for solving these semantic tasks. Finally, Lett-Fl was not significantly associated with the outcome of our semantic tasks (see Table 2 and Table 4). Given the assumed reliance of mPPT on control resources (on top of representational ones), this is an unexpected result, which casts doubt on the suitability of Lett-Fl for measuring control resources involved in (our) semantic tasks. We will take up this point later on in this section.

In a final step, we wanted to verify the external validity of the Cat/Free-Asymm index as a measure of control resources. To this end, we searched for an interaction between Cat/Free-Asymm and RT/ Word-freq in predicting mPPT and PN accuracy, respectively. The rationale of this investigation was as follows. Assuming that Cat/Free-Asymm is a measure of control function, participants with high values on this index (suggestive of control deficits) should be disproportionately impaired with task items requiring more executive control. As we suggested (see Introduction), such items are likely to be slow items of the mPPT task (since slow RT probably relates to a controlled, i.e., non-automatic, access of the relevant information) and high-frequency items in the PN task. In fact, it has been proposed that high-frequency words need a more controlled process for selecting the relevant meaning across competitors and that the uncontrolled activation of irrelevant meaning could generate interference during the naming process [4,18]. Both critical interactions were found to be statistically reliable (see Table 3B and Table 5B) and the relationship between variables was that expected according to our assumptions (see Figure 3A and Figure 4A), i.e., the performance gap between high and low scores on our index of control functioning increased with increasing RT (in mPPT) and word frequency (in PN).

In a final step, we wanted to verify whether Lett-Fl behaved like our index of control functioning (i.e., Cat/Free-Asymm) in the interaction with our item-specific measures of reliance on control resources (i.e., RT and Word-freq). As shown on Figure 3B and Figure 4B, the pattern of interaction was roughly similar; however, only the interaction with Word-Freq turned out to be statistically significant, whilst Lett-Fl did not reliably interact with RT. This result suggests that Cat/Free-Asymm might be a better index of control resources than Lett-FL. This could be the case for two reasons: (i) either because it is better suited for measuring control resources in these pathological populations, without the confounding of a possible trade-off effect between control and representational resources discussed above; (ii) or simply because it captures some kind of control function that is more involved than that indexed by Lett-Fl in (our) semantic tasks.

## 5. Conclusions

In conclusion, we want to propose some remarks on the potential relevance of the results discussed above and on the strengths and weaknesses of the present study.

From a clinical perspective, it is noteworthy that the F-Assoc task we presented here can be easily constructed in many languages, starting from already available associative norms, and their instructions are easy to comprehend also for people suffering from mental deterioration. According to our preliminary results, it seems that F-Assoc, jointly used with commonly administered verbal fluency tasks (Cat-FL and Lett-FL), provides a suitable tool for detecting qualitatively different profiles of semantic impairment in SD and AD, an achievement not yet fulfilled by any easily administrable battery of semantic tests. Should the suitability of F-Assoc for this purpose be confirmed in subsequent investigations, the clinical merits of this approach will be undeniable.

To achieve this, however, many issues still need to be addressed. First, these results need to be replicated in a bigger sample of people suffering from SD and AD, encompassing a broader range of disease severity. Second, the external validity of our representational and control indices (i.e., F-Assoc and Cat/Free-Asymm) need to be demonstrated, investigating its explanatory power in a wider array of semantic tasks (not only mPPT and PN). Finally, the relationship between Cat/Free-Asymm and other indices of control functions (including Lett-Fl) needs to be investigated in further research.

## Figures and Tables

**Table 1:** Mean (SD) of the demographic features across the experimental groups.

	SD	AD	NC
Gender (M/F)	6/4	5/5	8/17
Age (years)	64.1 (6.1)	74.1 (4.7)	73.6 (5.7)
Education (years)	13.4 (3.5)	8.6 (3.4)	11.0 (3.7)
MMSE	20.2 (5.4)	20.3 (4.6)	28.1 (1.4)

AD—Participants with Alzheimer’s disease, NC—normal controls, SD—participants with semantic dementia.

**Table 2:** Coefficients for mixed effects model contrasting the role of Group and Diagnostic tasks as unique predictors.

Predictor	*n*. AD/SD		Estimate	Predicted Probability Correct	−2LL	Diff-2LL(df)	*p*
Cat-Fl	Low 3/7	Intercept	1.2507	78%			
	High 7/3	High Cat-Fl	+1.2136	92%	902.8	10.223 (1)	0.001
Group		Intercept	1.2604	78%			
		AD	+1.1916	92%	903.5	9.4899 (1)	0.002
Free-Ass	Low 2/8	Intercept	1.3837	80%			
	High 8/2	High Free-Ass	+0.9456	91%	907.7	5.364 (1)	0.021
Lett-Fl	Low 4/6	Intercept	1.6134	83%			
	High 6/4	High Lett-Fl	+0.4906	89%	911.7	1.289 (1)	0.256

Of accuracy on mPPT. (−2LL) −2 × log likelihood of the model; (Diff−2LL(df)) difference in −2LL between tested model and baseline model.

**Table 3:** Coefficients for mixed effects model examining mPPT, taking multiple predictors simultaneously into account.

**(A) mPPT Accuracy as a Function of Group, Free-Associations and Cat-Fl**				
	**Estimate**	**SE**	***z***	**Pr (>|z|)**
Intercept	0.9459	0.0833	0.454	0.650
Age	−0.0074	0.0308	−0.241	0.810
Education	0.0139	0.0444	0.312	0.755
Group SD	−0.4906	0.4268	−1.150	0.250
Free-Ass	1.8134	1.6903	1.073	0.283
Cat-Fl	0.0578	0.0250	2.312	0.021
**(B) mPPT Accuracy as a Function of RT, Free-Associations and RT by Free-Ass Interaction**				
	**Estimate**	**SE**	***z***	**Pr (>|z|)**
Intercept	2.92018	1.04406	2.797	0.005
RT	−105919	0.36974	−2.865	0.004
Free-Ass	9.05794	1.85618	4.880	<0.001
RT: Free/Cat-Asym	−0.19492	0.06365	−3.062	0.002

**Table 4:** Coefficients for mixed effects model contrasting the roles of Group and Diagnostic tasks as unique predictors of accuracy in PN.

Predictor	*n*. AD/SD		Estimate	Predicted Probability Correct	−2LL	Diff−2LL(df)	*p*
Group		Intercept	0.1770	54%			
		AD	+3.3478	97%	705.83	15.434 (1)	0.001
Free-Ass	Low 8/2	Intercept	0.5010	62%			
	High 2/8	High Free-Ass	+2.9921	97%	710.36	10.906	0.001
Cat-Fl	Low 7/3	Intercept	0.06182	65%			
	High 3/7	High Cat-Fl	+2.7342	97%	712.65	8.6143	0.003
Lett-Fl	Low 6/4	Intercept	1.1184	75%			
	High 4/6	High Lett-Fl	+1.4538	93%	719.16	2.1026	0.147

(−2LL) −2 × log likelihood of the model; (Diff−2LL(df)) difference in −2LL between tested model and baseline model.

**Table 5:** Coefficients for mixed effects model examining PN, taking multiple predictors simultaneously into account.

**(A) PN Accuracy as a Function of Group, Free-Associations and Cat-Fl**				
	**Estimate**	**SE**	***z***	**Pr (>|z|)**
Intercept	−4.6501	2.8083	−1.656	0.098
Age	0.0451	0.0416	1.085	0.278
Education	−0.0261	0.0566	−0.461	0.645
Group SD	−0.5615	0.5475	−1.026	0.305
Free-Ass	7.5487	2.1638	3.489	<0.001
Cat-Fl	0.1187	0.0342	3.477	<0.001
**(B) PN Accuracy as a Function of W-freq, Free-Associations and W-freq by Free-Ass Interaction**				
	**Estimate**	**SE**	***z***	**Pr (>|z|)**
Intercept	−7.4580	1.3509	−5.5121	<0.001
W-freq	2.2038	0.5739	3.840	<0.001
Free-Ass	27.6789	2.9596	9.352	<0.001
W-freq: Free/Cat-Asym	−0.5546	0.1013	−5.476	<0.001

## Data Availability

The datasets generated during and/or analysed during the present study are available on request from the corresponding author.

## References

[1] Zannino G.D., Caltagirone C., Carlesimo G.A. (2015). The contribution of neurodegenerative diseases to the modelling of semantic memory: A new proposal and a review of the literature. Neuropsychologia.

[2] Fodor J.A. (1983). The Modularity of Mind—An Essay on Faculty Psychology.

[3] Nebes R.D. (1989). Semantic memory in Alzheimer’s disease. Psychol. Bull..

[4] Rogers T.T., Patterson K., Jefferies E., Ralph M.A.L. (2015). Disorders of representation and control in semantic cognition: Effects of familiarity, typicality, and specificity. Neuropsychologia.

[5] MacKenzie I.R.A., Neumann M., Bigio E.H., Cairns N.J., Alafuzoff I., Kril J., Kovacs G.G., Ghetti B., Halliday G., Holm I.E. (2008). Nomenclature for neuropathologic subtypes of frontotemporal lobar degeneration: Consensus recommendations. Acta Neuropathol..

[6] Gorno-Tempini M.L., Hillis A.E., Weintraub S., Kertesz A., Mendez M., Cappa S.F., Ogar J.M., Rohrer J.D., Black S., Boeve B.F. (2011). Classification of primary progressive aphasia and its variants. Neurology.

[7] Snowden J.S., Goulding P.J., Neary D. (1989). Semantic dementia: A form of circumscribed cerebral atrophy. Behav. Neurol..

[8] Rogers T.T., Ivanoiu A., Patterson K., Hodges J.R. (2006). Semantic memory in Alzheimer’s disease and the frontotemporal dementias: A longitudinal study of 236 patients. Neuropsychology.

[9] Corbett F., Jefferies E., Burns A., Ralph M.A.L. (2015). Deregulated semantic cognition contributes to object-use deficits in A lzheimer’s disease: A comparison with semantic aphasia and semantic dementia. J. Neuropsychol..

[10] Henry J.D., Crawford J.R. (2004). A Meta-Analytic Review of Verbal Fluency Performance Following Focal Cortical Lesions. Neuropsychology.

[11] Henry J.D., Crawford J.R., Phillips L.H. (2004). Verbal fluency performance in dementia of the Alzheimer’s type: A meta-analysis. Neuropsychologia.

[12] Adlam A.-L.R., Patterson K., Bozeat S., Hodges J.R. (2010). The Cambridge Semantic Memory Test Battery: Detection of semantic deficits in semantic dementia and Alzheimer’s disease. Neurocase.

[13] Zannino G.D., Perri R., Teghil A., Caltagirone C., Carlesimo G.A. (2018). Associative Agreement as a Predictor of Naming Ability in Alzheimer’s Disease: A Case for the Semantic Nature of Associative Links. Front. Behav Neurosci..

[14] Howard D., Patterson K. (1992). Pyramids and Palm Trees: A Test of Semantic Access from Pictures and Words.

[15] Marczinski C.A., Kertesz A. (2006). Category and letter fluency in semantic dementia, primary progressive aphasia, and Alzheimer’s disease. Brain Lang..

[16] Perry R.J., Watson P., Hodges J.R. (2000). The nature and staging of attention dysfunction in early (minimal and mild) Alzheimer’s disease: Relationship to episodic and semantic memory impairment. Neuropsychologia.

[17] Catricalà E., Della Rosa P.A., Ginex V., Mussetti Z., Plebani V., Cappa S. (2012). An Italian battery for the assessment of semantic memory disorders. Neurol. Sci..

[18] Hoffman P., Rogers T.T., Ralph M.A.L. (2011). Semantic Diversity Accounts for the “Missing” Word Frequency Effect in Stroke Aphasia: Insights Using a Novel Method to Quantify Contextual Variability in Meaning. J. Cogn. Neurosci..

[19] Measso G., Cavarzeran F., Zappalà G., Lebowitz B.D., Crook T.H., Pirozzolo F.J., Amaducci L.A., Massari D.C., Grigoletto F. (1993). The mini-mental state examination: Normative study of an Italian random sample. Dev. Neuropsychol..

[20] McKhann G.M., Knopman D.S., Chertkow H., Hyman B.T., Jack C.R., Kawas C.H., Klunk W.E., Koroshetz W.J., Manly J.J., Mayeux R. (2011). The diagnosis of dementia due to Alzheimer’s disease: Recommendations from the National Institute on Aging-Alzheimer’s association workgroups on diagnostic guidelines for Alzheimer’s disease. Alzheimers Dement..

[21] Dell’Acqua R., Lotto L., Job R. (2000). Naming times and standardized norms for the italian PD/DPSS set of 266 pictures: Direct comparisons with American, English, French, and Spanish published databases. Behav. Res. Methods Instrum. Comput..

[22] R Core Team (2020). R: A Language and Environment for Statistical Computing.

[23] Crawford J.R., Garthwaite P.H., Porter S. (2010). Point and interval estimates of effect sizes for the case-controls design in neuropsychology: Rationale, methods, implementations, and proposed reporting standards. Cogn. Neuropsychol..

[24] Bates D., Mächler M., Bolker B., Walker S. (2015). Fitting Linear Mixed-Effects Models Using lme4. J. Stat. Softw..

[25] Carlesimo G., Caltagirone C., Gainotti G., Fadda L., Gallassi R., Lorusso S., Marfia G., Marra C., Nocentini U., Parnetti L. (1996). The Mental Deterioration Battery: Normative Data, Diagnostic Reliability and Qualitative Analyses of Cognitive Impairment. Eur. Neurol..

[26] Lüdecke D. (2018). ggeffects: Tidy Data Frames of Marginal Effects from Regression Models. J. Open Source Softw..

[27] Jaeger T.F. (2008). Categorical data analysis: Away from ANOVAs (transformation or not) and towards logit mixed models. J. Mem. Lang..

[28] Barr D.J., Levy R., Scheepers C., Tily H.J. (2013). Random effects structure for confirmatory hypothesis testing: Keep it maximal. J. Mem. Lang..

